# Predicting combinations of drugs by exploiting graph embedding of heterogeneous networks

**DOI:** 10.1186/s12859-022-04567-4

**Published:** 2022-01-11

**Authors:** Fei Song, Shiyin Tan, Zengfa Dou, Xiaogang Liu, Xiaoke Ma

**Affiliations:** 1 School of Computer Science and Technology, Xidian University, No. 2 South TaiBai Road, 710071 Xi’an, People’s Republic of China; 2 The 20-th Research Institute, China Electronics Technology Group Corporation, Guanhua Road, 710071 Xi’an, People’s Republic of China; 3 School of Mathematics and Statistics, Northwestern Polytechnical University, No. 1 Dongxiang Road, 710072 Xi’an, People’s Republic of China

**Keywords:** Drug combination, Heterogeneous network, Graph embedding, Joint learning

## Abstract

**Background:**

Drug combination, offering an insight into the increased therapeutic efficacy and reduced toxicity, plays an essential role in the therapy of many complex diseases. Although significant efforts have been devoted to the identification of drugs, the identification of drug combination is still a challenge. The current algorithms assume that the independence of feature selection and drug prediction procedures, which may result in an undesirable performance.

**Results:**

To address this issue, we develop a novel *Se*mi-supervised *H*eterogeneous *N*etwork *E*mbedding algorithm (called SeHNE) to predict the combination patterns of drugs by exploiting the graph embedding. Specifically, the ATC similarity of drugs, drug–target, and protein–protein interaction networks are integrated to construct the heterogeneous networks. Then, SeHNE jointly learns drug features by exploiting the topological structure of heterogeneous networks and predicting drug combination. One distinct advantage of SeHNE is that features of drugs are extracted under the guidance of classification, which improves the quality of features, thereby enhancing the performance of prediction of drugs. Experimental results demonstrate that the proposed algorithm is more accurate than state-of-the-art methods on various data, implying that the joint learning is promising for the identification of drug combination.

**Conclusions:**

The proposed model and algorithm provide an effective strategy for the prediction of combinatorial patterns of drugs, implying that the graph-based drug prediction is promising for the discovery of drugs.

## Background

Drug discovery is critical for the therapy of complex diseases, particularly for these scare diseases such as cancers [1]. The current algorithms for drug discovery are roughly classified into two classes, i.e., the bio-chemical and computational strategies. The traditional strategy makes full use of chemical and biological experiments to synthesize the novel drugs and validate the toxicity of drugs. Generally, this strategy is reliable and effective, whereas it is also criticized for the time and finance. Usually, the design of a drug takes more than 10 years with billions of dollars. Therefore, there is a critical need for alternatives for the traditional method. The traditional assumption that drugs can only be applied to specific diseases, which limits the applications of drugs [2]. Actually, drugs for a specific disease may also be a potential alternative for other diseases because some complex diseases with the same or similar underlying mechanisms, requiring the same or similar therapy strategies and drugs [3].

The accumulated identified drugs and corresponding features provide an opportunity for the identification of potential drugs for diseases without drugs for therapy, which significantly alleviates the burden of period and finance. Furthermore, the predicted drug patterns possibly shed light on the products of drugs. On the basis of the assumption that similar drugs have the same or similar performance on therapy, great efforts have been devoted to this issue with an immediate purpose to identify the potential drugs for diseases by exploiting the similarity among drugs [4]. The major difference between these algorithms lies in how to define and infer the similarity by exploiting various features, such as structure, toxicity, and so on. Given the target disease and potential drugs, the vast majority of current algorithms focus on the ranking of potential drugs by measuring the similarity between the known drugs and candidates, ignoring the effects of drugs combination. Actually, the combination of drugs promotes the performance of treatment. For example, the combination of BRAF and V600E significantly improves the therapy of melanoma, and many drug combinations are approved by FDA (Food and Drug Adminstration) [5].

Therefore, it is promising for the identification of combinatorial drugs for diseases. But, it is highly non-trivial to predict the combination of drugs because combinatorial drugs are very likely to produce synergistic, additive, antagonistic, or even suppressive effects [6]. Consequently, the antagonistic or suppressive drug-drug interactions lead to undesirable consequences. For example, drug DDIs, accounting for 3–5% of inpatient medication errors, often lead to patient morbidity and mortality [7–9]. Even though it is difficult, great efforts have been devoted to the identification of combination drugs largely due to the merits of applications [10–12].

Specifically, the most reliable and intuitive strategy for the identification of drug combinations is biochemical experiments, including the typical P450 testing [13] and transporter-associated interactions [14]. Compared to drug discovery, the identification of drug combinations based on biochemical is much more complicated for several reasons. First, the factors involved in drug combinations, such as the positive and negative interactions among drugs, are much more than the traditional single drug discovery, which imposes a great challenge on the design of experiments and protocols since the balance of multiple factors is complicated. Second, the biochemical experiment-based approaches in clinical trials usually are criticized for the expensive cost, intolerable duration, and unpredictable effects of clinical validation. Finally, the selection of candidates of drug combination is usually impractical because the solution space exponentially increases.

Therefore, many computational algorithms have been devoted to the identification of drug combinations to alleviate the burden of biochemical methods by exploiting the similarity of various drugs [15, 16]. On the basis of the computational strategies, the available algorithms are classified into three classes: statistic-, feature- and network-based methods. The most intuitive and straightforward statistic-based methods are the Loewe additivity and Bliss independence models [17], which are two commonly used methods for the quantification of synergy between drug combinations. Specifically, the Loewe additivity model assumes that drugs can be combines if the inhibitors have the same or similar mechanism of regulations, whereas Bliss makes use of independence assumption based on probability theory [18]. The advantage of statistic-based methods is simple and easy to implement. However, these approaches are criticized for two limitations. First, the prerequisite of statistic strategy is the large or super-large scale samples to guarantee the accuracy of prediction, which hampers the application of these algorithms because some drugs cannot be validated on the huge population. Second, the accuracy of prediction is not desirable because the statistic strategy only focuses on the significance of the difference between groups with various responses, neglecting the features of drugs.

To overcome these problems, the feature-based methods predict the combination of drugs by exploiting the machine learning techniques, such as classification, which aim to extract the most discriminative features. The major difference of feature-based algorithms depends on how to select the features of drugs, and what classifiers to choose for prediction. For example, PDC-SGB [19] integrates six types of features to predict drug combinations, including the 2-dimensional molecular structures, structural similarity, anatomical therapeutic similarity, protein–protein interaction, chemical–chemical interaction, and disease pathways, where three classification algorithms to build the drug combination prediction models are proposed. Compared to the statistic-based methods, PDC-SGB not only significantly improves the accuracy of prediction of the drug combination, but also ranks the importance of features. Sun et al. [20] predict drug combinations by integrating the gene expression data of multiple drugs, which enhances the performance of algorithms, indicating that gene expression is also a discriminative feature for drug combinations. To validate the role of genomic features, HNAI [21] fuses the drug phenotypic, therapeutic, structural, and genomic similarities to the prediction of drug combinations by using five machine learning-based classifiers.

Even though the feature-based algorithms dramatically outperform the statistic-based methods, the performance is still unsatisfied because the relations among features are ignored, failing to characterize the indirect relations among features. Fortunately, networks (also called graphs) provide an effective and efficient manner to model and characterize complex systems, where vertices denote entities and edges represent interactions among vertices [22, 23]. Thus, many algorithms have been developed by utilizing the networks of features with an immediate purpose to improve the performance of prediction of drug combinations by exploring the indirect relations of features. The key techniques involved in these algorithms concentrate on the network construction and analysis, where network construction determines how to model the features by using similarity of features, and network analysis focus on how to extract the indirect relations from networks to facilitate the prediction of drug combinations. For example, Liu et al. [24] construct a heterogeneous network by exploiting the similarity between drug and protein, in which three types of similarity, i.e., drug–drug, drug–protein, and protein–protein, are integrated. Then, they perform random walk to extract features of drugs by exploiting the topological structure of the constructed network, which serves as the input of a gradient tree boosting (GTB) classifier to predict drug combinations. To further explore features, NDD [25] employs a nonlinear fusion method for multiple types of similarity to achieve high-level features, and then predicts drug combinations by using the neural network. Li et al. [26] integrate multiple types of features to improve the accuracy of prediction by using the neighbor recommender strategy in networks. NIMS [27] made use of networks to screen potential drug combinations, where the disease-specific biological network is treated as a therapeutic target.

To further improve the performance of prediction, integrating features of various entities is also promising. For example, NLLSS [28] predicts the potential synergistic drug combinations by integrating different kinds of information, such as known synergistic drug combinations, drug-target interactions, and drug chemical structures, which are integrated into a heterogeneous network. Cheng et al. [29] develop a comprehensive drug-drug interaction network incorporating 6946 interactions of 721 approved drugs using data from DrugBank by using the phenotypic similarity, therapeutic similarity, chemical structure similarity, and gene similarity. EPSDC [30] utilizes the ensemble method to predict the drug combinations by integrating multiple-sources information, where construct a feature vector for each pair of drugs by exploiting the drug similarity. Then, the rank of drug pairs is performed by analyzing the topological structure of the heterogeneous drug–target network. Finally, EPSDC fulfills the prediction of drug combination by balancing the rank and output of feature-based classifiers.

Even though significant efforts have been devoted to the prediction of drug combinations, many unsolved problems remain. For example, the current network-based algorithms are time-consuming, hindering the applications of large-scale networks. Furthermore, the accuracy of current methods can be further improved. Finally, most network-based algorithms construct the heterogeneous network to characterize the interactions among drug and gene/protein. However, these algorithms extract features of drugs by using the topological analysis strategy for homogeneous networks, ignoring heterogeneity of networks. Recently, graph embedding has been applied to heterogeneous networks, aiming to learn features by preserving the topological structure [31–39]. Different from homogeneous networks, it is challenging to develop methods for modeling the heterogeneous types of vertices and edges in a unified way. Heterogeneous information network embedding aims to obtain the low-dimensional representation for each vertex by preserving the topological structure of networks. For example, PME [34] utilizes the metric learning to simultaneously preserve the first- and second-order proximity of heterogeneous networks. Dong et al. [35] design the meta-path-based random walks to neighborhoods of vertices and then leverages a skip-gram model to perform embedding. metapath2vec preserves both the structures and semantics of a given heterogeneous network by simultaneously learning the low-dimensional and latent embedding for vertices. SHINE [38] extracts the latent representations of vertices by preserving the signs of edges by using auto-encoder, and there are some deep learning based methods [39]. In this study, we investigate the possibility of predicting drug combinations by fully exploiting the graph embedding in heterogeneous networks, which is one of the major motivations.

To overcome these problems, we develop a semi-supervised heterogeneous network embedding algorithm (called SeHNE) to identify drug combination by integrating features of drugs and proteins, which consists of three major components, i.e., network construction, graph embedding for heterogeneous networks, and prediction of drug combinations. To construct the heterogeneous network for drugs, we integrate the drug–drug, protein–protein interactions, and drug–target associations. The graph embedding for drugs is performed by nonnegative matrix factorization for the drug-drug and drug–target networks, where the basis matrices are fused to generate the heterogeneous features for drug pairs. To incorporate protein–protein interaction network into feature extraction, we employ the regularization strategy, where the local topological structure of proteins are preserved. Finally, the feature extraction of heterogeneous and prediction of drug combination are jointly learned. In this case, the features are extracted under the guidance of the classifier, thereby improving the discriminative of features. The experimental results demonstrate that the proposed algorithm outperforms state-of-the-art methods in terms of various measurements, such as the area under curve (AUC), average precision (AP), and accuracy.

**Fig. 1:**
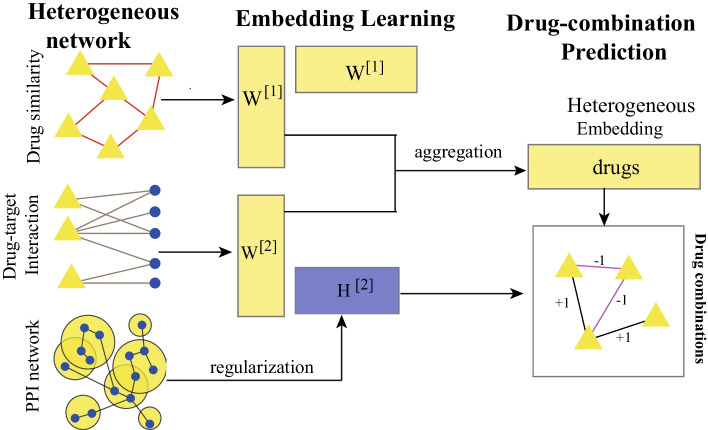
The overview of the proposed algorithm, which consists of two major components, i.e., heterogeneous embedding for drug, and drug combination prediction, where graph embedding learns the features of drugs by using matrix factorization for drug combination prediction, and drug combination prediction performs the classification

## Results

### Overview of SeHNE

The overview of SeHNE is depicted in Fig. 1, which consists of three major components, i.e., graph embedding in heterogeneous network, and prediction of drug combination. SeHNE jointly learns features of drugs and prediction of drug combination, where graph embedding for heterogeneous networks is selected to obtain features of drugs, and SVM [40] is used for the prediction of drug combination.

To check whether SeHNE is sensitive to the selection of classifier, six typical classifiers, including k-nearest neighbor (KNN), logistic regression (LR), random forest (RF) [41], gradient boosting tree (GBT) [12], adaboost (ADB) [42] and linear discriminative analysis (LDA) [43], are selected to replace SVM. The performance of SeHNE with various classifiers in terms of different measurements, including accuracy, precision, recall, F-measure, MCC, and AUC, is shown in Table 2, where SeHNE obtains a similar performance, implying SeHNE is not sensitive to classifiers.

To check whether SeHNE is sensitive to different similarities, we replace Anatomical Therapeutic Chemical(ATC) with chemical structure similarity (CSS) (Methods Section). The performance of SeHNE is shown in Table 1, where SeHNE is also stable. These results demonstrate that SeHNE is not sensitive to similarity.

**Table 1:** Comparison of various similarity strategies

Similarity	Accuracy	Precision	Recall	F-measure	MCC	AUC
ATC	0.751	0.566	0.635	0.751	0.436	0.753
CSS	0.750	0.568	0.618	0.750	0.431	0.758
ATC and CSS	0.755	0.560	0.587	0.750	0.427	0.749

**Table 2:** Performance of various compared algorithms with different classifiers

Classifier	Accuracy	Precision	Recall	F-measure	MCC	AUC
SVM	0.751	0.566	0.635	0.751	0.436	0.753
KNN	0.736	0.471	0.489	0.727	0.371	0.673
LDA	0.743	0.486	0.540	0.738	0.397	0.691
ADB	0.746	0.490	0.540	0.741	0.404	0.693
GBT	0.741	0.480	0.516	0.734	0.387	0.683
RF	0.732	0.465	0.486	0.723	0.361	0.668
LR	0.741	0.479	0.510	0.733	0.385	0.681

### Parameter analysis

**Fig. 2:**
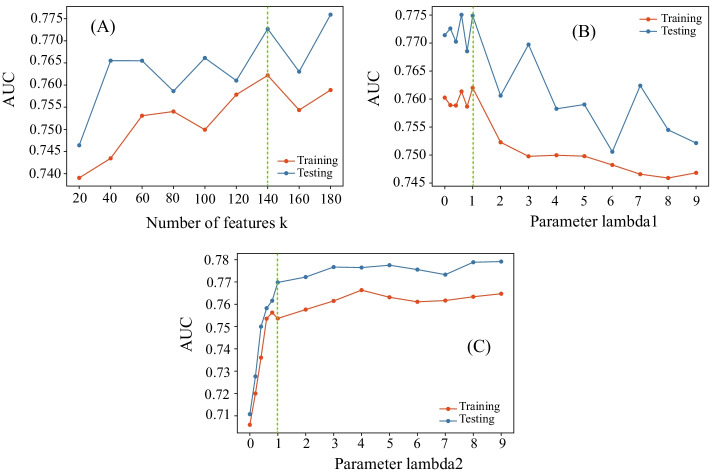
Parameter effects in terms of AUC: **A** AUC versus *k*, **B** AUC versus $\lambda _{1}$, and **C** AUC versus $\lambda _{2}$

There are three parameters involved in SeHNE, where parameter $\lambda _{1}$ and $\lambda _{2}$ control the importance of drug-target network and classifier, and *k* denotes the number of features in graph embedding. We investigate how AUC of SeHNE changes by varying the value of one parameter with values of other parameters fixed. The drug combination data is split into the training and testing data. We use training data for the 10-fold cross-validation to obtain AUC of SeHNE and then utilize training data to construct the model and testing data to measure the accuracy. How AUC of SeHNE changes as parameter *k* varying from 20 to 180 by fixing $\lambda _{1}$ and $\lambda _2$ as 1 is shown in Fig. 2A. As *k* increases from 20 to 140, the accuracy of SeHNE also improves. However, the performance decreases as *k* keeps increasing. When *k* is small, the features in embedding are insufficient to characterize drugs. When *k* is large, the redundancy of features results in undesirable performance. When *k* equals 140, SeHNE achieves the best performance.

By fixing *k*=140, and $\lambda _{2}$=1, how AUC of SeHNE changes as parameter $\lambda _{1}$ increases from 0 to 9 with a gap 1 is shown in Fig. 2B, where the AUC decreases as $\lambda _{1}$ increases. The possible reason is that, when $\lambda _{1}$ is small, the drug–target and drug networks reach a good balance. When $\lambda _{1}$ is large, the drug–target network dominates the objective function, where features deviate from the drug network. SeHNE obtains the best performance at $\lambda _{1}$ = 1. Figure 2C shows the AUC of SeHNE by varying $\lambda _{2}$ from 0 to 9 by setting *k* = 140, and $\lambda _{1}$ = 1. As $\lambda _{2}$ increases from 0 to 1, the performance improves and then keeps stable. Therefore, in the forthcoming experiments, we set $\lambda _1=\lambda _2=1$, and $k=140$.

**Fig. 3:**
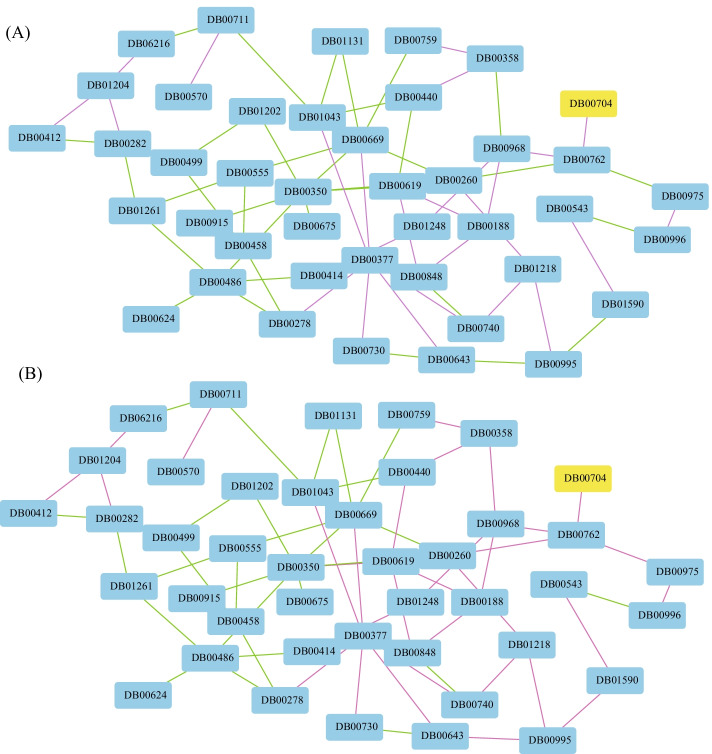
A schematic example of drug combinations: **A** the ground truth combination of drugs, and **B** the prediction combinatorial patterns of drugs, where green edges represent antagonistic DDIs, and purple ones denotes synergistic relationship

### Performance on drug combination prediction

Before presenting the detailed performance of various algorithms, we give an illustrative example as shown in Fig. 3, where panel A is the benchmark interactions, and panel B is the predicted DDI of drugs. In Fig. 3, colors correspond to the type of interactions among drugs, where the green edges denote antagonistic, and purple edges represent the synergistic relationship. It is obvious the predicted interactions among drugs are highly consistent with the ground truth ones, i.e., the accuracy is 0.7553 on test data. Figure 3 demonstrates that the proposed algorithm is efficient for the prediction of drug combination (Table 2).

**Fig. 4:**
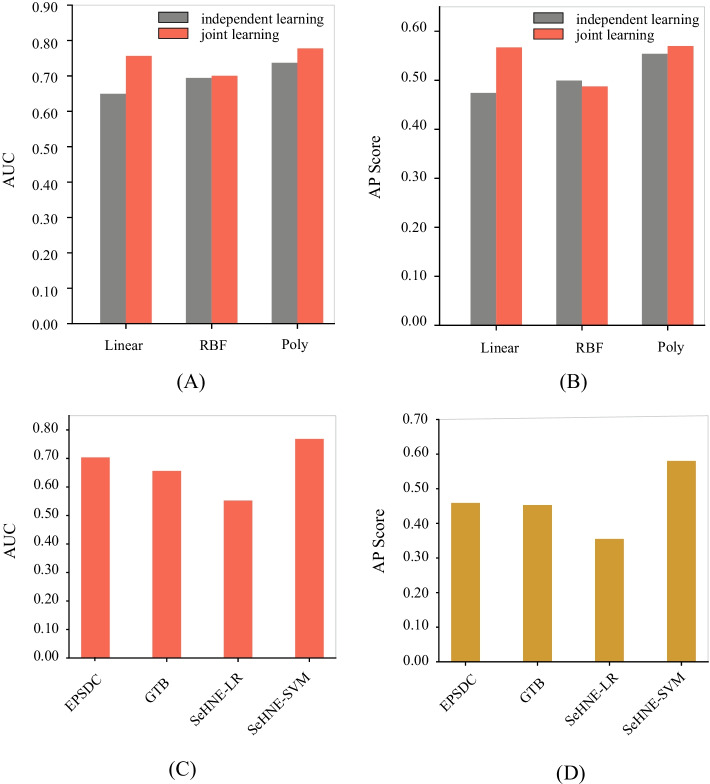
The performance of SeHNE by using various kernel functions for SVM, and the performance of various algorithms on the prediction of drug combination

In SeHNE, we adopt SVM as the classifier to predict drug combinations. To select the best kernel function for SVM, we compare SeNMF by using various kernel functions, including the linear, logistic regression, and RBF kernels. The AUC and AP scores of SeHNE by using various kernel functions are shown in Fig. 4, where panel A is for AUC, and B for average precision score (AP score). These panels show that the polynomial kernel significantly outperforms the others on AUC and AP score. However, the effect of SeHNE with logistic regression is not ideal because the feature space is large. Thus, we select the polynomial kernel in the experiments.

In this study, the graph embedding and prediction are jointly learned. It is natural to ask whether joint learning is promising for the drug combination. We execute SeHNE in two different strategies, i.e., independent learning and joint learning, where independent learning first extracts the graph embedding and then utilizes SVM to predict drub combination. The results are shown in Fig. 4A, B, where joint learning is superior to independent learning in terms of AUC and AP scores. These results demonstrate that joint learning is promising for the drug combination.

Finally, we compare SeHNE with state-of-the-art to fully validate the performance of various algorithms. Two algorithms are selected for a comparison, including GTB [24] and EPSDC [30]. These algorithms are selected because EPSDC is the newest method that is simultaneously fusing heterogeneous network and ATC similarity. GTB is the typical algorithm drug combination. The 10-fold cross-validation strategy is used to testify the performance of various algorithms. The AUC and AP score are used to quantify the performance of algorithms. The results are shown in Fig. 4, where panel C is for AUC and panel D is for AP score.

The result demonstrates that the SeHNE algorithm outperforms the others in terms of AUC score and AP score. The SVM is better than logistic regression for SeHNE. The reason is that SVM is more discriminative since it exploits the critical features for the prediction of the drug combination. There are three reasons why the proposed algorithm outperforms state-of-the-art methods: SeHNE extracts features of drugs by exploiting the heterogeneous network, which is more discriminative than current algorithms because the indirect relations are explored. Joint learning improves the quality of features since features are selected under the guidance of classification. Matrix factorization extracts the latent features from heterogeneous networks, which is more comprehensive to depict drug combinations. These results demonstrate that joint learning of heterogeneous networks and classification is promising for the prediction of drug combinations.

**Fig. 5:**
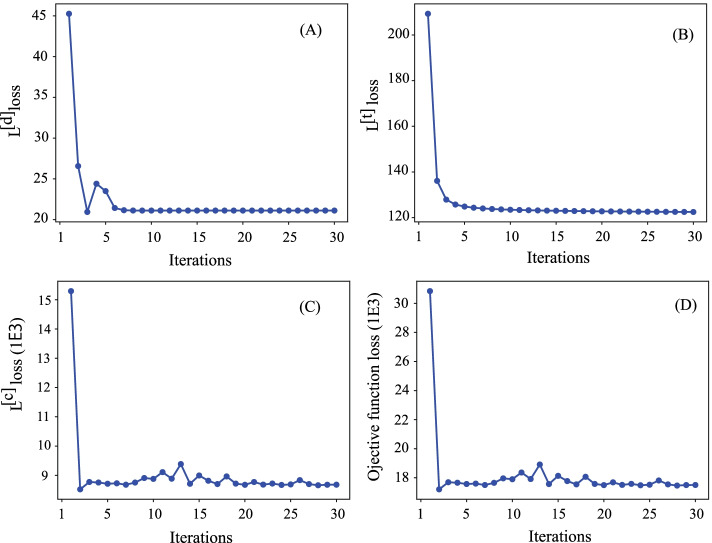
Convergence analysis of SeHNE with various strategies: **A** ${\mathcal {L}}^{[d]}$ loss, **B** ${\mathcal {L}}^{[t]}$ loss, **C** ${\mathcal {L}}^{[c]}$ loss, and **D** objective function loss versus the number of iterations

### Convergence analysis

The proposed algorithm consists of multiple stages, where the objective function is composed of several components, i.e., ${\mathcal {L}}^{[d]}$ loss, ${\mathcal {L}}^{[t]}$ loss, and ${\mathcal {L}}^{[c]}$ loss. To validate the convergence of the proposed algorithm, we check those components changes as the number of iterations changes as shown in Fig. 5, where panel A is for ${\mathcal {L}}^{[d]}$ loss, B for ${\mathcal {L}}^{[t]}$ loss, and C for ${\mathcal {L}}^{[c]}$ loss, respectively. From these panels, it is easy to conclude that these sub-procedures quickly converge, i.e., they only take 30 iterations to converge.

Finally, we investigate how the objective function of the proposed algorithm changes as the number of iterations, which is shown in Fig. 5 D. SeHNE converges within 30 iterations, implying that the proposed algorithm is efficient. There are two reasons why the proposed algorithm quickly converges. First, SeHNE factorizes the drug–target network by regularizing the drug–drug and protein–protein interaction (PPI) networks, which enhances the efficiency of feature extraction. Second, the heterogeneous features of proteins and drugs serve as prior information, which accelerates the speed of convergence.

## Discussion

Drug discovery is critical for the therapy of complex diseases, particularly for these scare cancers. However, the biological experiment-based methods are time and finance consuming, requiring efficient and effective alternatives for this issue. And, the computational approaches provide an alternative for the traditional bio-chemical strategy by exploiting features of various entities, such as genes, and proteins. Even though great efforts have been devoted to this issue, vast majority of algorithms solely focus on the identification of potential drugs for complex diseases based on the assumption that similar drugs have similar functions. Therefore, current algorithms concentrate on how to define and compute similarity among drugs with various strategies.

Actually, drug combination is also critical needed since therapy of cancers is complicated, where a single drug is insufficient. However, effort for drug combination is really limited largely because the identification of drug combination is much more complicated than the detection of similar drugs. In this study, we present a novel integrative method for drug combination, where drugs, proteins, and interactions are integrated into a heterogeneous network. The proposed algorithm jointly learns the graph embedding and classification. On the one hand, similar to the previous work [24–30], SeHNE fuses the drug-drug networks, drug-protein networks, and protein-protein networks into a heterogeneous network and extracts interesting feature for each drug combination from the heterogeneous network. Furthermore, similar to [24, 25], SeHNE takes features of combined drugs as input, and adopts SVM to predict drug combination. On the other hand, different from these works [34, 36, 38], SeHNE joins the procedures of feature extraction and prediction, where matrix factorization is employ to obtain graph embedding as features of drugs.

SeHNE outperforms baselines in terms of accuracy, implying that the joint learning strategy is more accuracy to model and characterize drug combination. There are two reasons explain why the superiority of the proposed algorithm. First, the topological structure of heterogeneous networks provides complemental information for drugs, thereby improving the quality of features of drugs. Second, graph embedding reflects the latent features of drugs by preserving structural information of drugs.

## Conclusion

A novel algorithm for the prediction of combination of drugs is proposed, where multiple types entities are integrated to construct heterogeneous networks. Compared with state-of-the-art methods, the proposed algorithm fully makes use of the indirect relations among various entities, which provides a better way to characterize the features of drugs. Furthermore, we present joint learning for feature extraction and prediction of drug combinations, where the features of drugs are more discriminative, resulting in an improved performance. The experimental results demonstrate that the proposed methods outperform the current algorithms in terms of accuracy.

Even though the proposed algorithm algorithm is promising for predicting combination of drugs, there are still some unsolved problems for further study, which are listed as

- In this study, the proposed algorithm only focuses on the combination of drug pairs, rather than the high-order combination, because the space of candidates for combinations of drugs exponentially increases. How to narrow the space of feasible drug combination is the foundation for the exploitation of high-order combination of drugs. The strategy for selecting candidates for high-order combination of drugs is critically needed.
- The developed algorithm makes use of the topological structure of heterogeneous networks to extract features of drugs, ignoring the intrinsic features of drugs. How to assign attributes, such as the structure and function of drugs to drugs is also promising for modeling and characterization of drugs.
- SeHNE only integrates the information of drugs and proteins without considering the regulation principle of drugs. How to integrate gene expression, drug responses and immune micro-environment are also interesting for the identification of drug combinations.

**Fig. 6:**
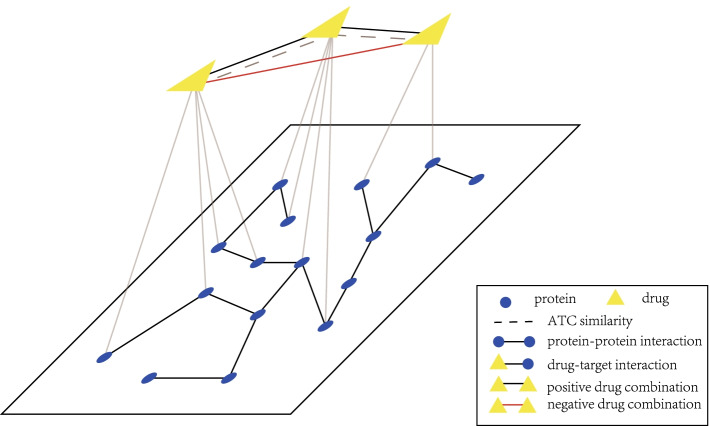
The schematic example of heterogeneous network for drug and proteins, which consists of four types of interactions, i.e., protein–protein interaction, ATC similarity, drug–target association, and known drug combinations

## Methods

In this section, we present the model, procedure, and analysis of the proposed algorithm.

### Notations

Before presenting the procedure of SeHNE, we present the notations and formulation of drug combination that are widely used in the forthcoming sections.

Given a group of vertices $\{v_{1},\ldots ,v_{n}\}$ (*n* is the number of vertices), a network is denoted by $G=(V,E)$, where $E=\{(v_{i},v_{j})\}$ is edge set. The adjacent matrix of *G* is represented by $A=(a_{ij})_{n\times n}$ where $a_{ij}$=1 if vertex $v_{i}$ and $v_{j}$ are connected by an edge, 0 otherwise. A network *G* is heterogeneous if and only if there are more than one type of vertices in *G*. For example, there are two types of vertices in the heterogeneous network in Fig. 6, where the yellow triangle vertices denote drug, and the blue circle ones are proteins. And, there are three types of interactions, i.e., drug–drug interactions, drug–protein interactions and protein–protein interactions.

Formally, let $V^{[d]}=\{v_{1}^{[d]},\ldots ,v_{n_d}^{[d]}\}$, and $V^{[p]}=\{v_{1}^{[p]},\ldots ,v_{n_{p}}^{[p]}\}$ be the drug, and protein set, respectively. And, $n_{d}$ and $n_{p}$ are the number of drugs and proteins, respectively. Among proteins, some proteins are targets of drugs, denoted by $V^{[t]}=\{v_{1}^{[t]},\ldots ,v_{n_{t}}^{[t]}\}$, where $V^{[t]}\subset V^{[p]}$. There are three types of interactions, including drug–drug, drug–target (proteins), and protein–protein interaction, denoted by $E^{[d]}=\{(v_{i}^{[d]},v_{j}^{[d]})\}$, $E^{[t]}=\{(v_{i}^{[d]},v_{j}^{[t]})\}$, $E^{[p]}=\{(v_{i}^{[p]},v_{j}^{[p]})\}$, respectively. For a sake of convince, we use $G^{[d]}=(V^{[d]},E^{[d]})$, $G^{[t]}=((V^{[d]},V^{[t]}),E^{[t]})$, and $G^{[p]}=(V^{[p]},E^{[p]})$, respectively. For the drug network $G^{[d]}$, the similarity matrix $S^{[d]}=(s_{ij}^{[d]})\in$[0,1] is constructed, where $s_{ij}^{[d]}$ represents the similarity between $v_{i}^{[d]}$ and $v_{j}^{[d]}$ in terms of ATC. The incidence matrix for $G^{[t]}$ is denoted by $B^{[t]}=(b_{ij}^{[t]})_{n_{d}\times n_{t}}$, where $b_{ij}^{[t]}$=1 if protein $v_{j}^{[t]}$ is the target of drug $v_{i}^{[d]}$, 0 otherwise. The adjacent matrix for $G^{[p]}$ is constructed as $A^{[p]}=(a_{ij}^{[p]})_{n_{p}\times n_{p}}$ with element $a_{ij}^{[p]}$ as 1 if an interaction between protein $v_{i}^{[p]}$ and $v_{j}^{[p]}$ exists, 0 otherwise.

### Problem definition

Given the heterogeneous network $G=(G^{[d]},G^{[t]},G^{[p]})$, $G^{[d]}$ denotes drug–drug similarity graph, $G^{[t]}$ denotes drug–target (protein) interaction graph, $G^{[p]}$ denotes protein–protein interaction graph. Drug combination aims to construct a prediction function $\phi$ to predict drug–drug interaction between drug $v_{i}^{[d]}$ and $v_{j}^{[d]}$. The prediction function $\phi$ is defined as

1 $$\begin{aligned} \phi : (v_{i}^{[d]},v_{j}^{[d]}) \longmapsto \{-1,0,+1\}, \end{aligned}$$

where − 1 denotes that the two drugs cannot be combined, and this drug combination may produce an antagonistic or even suppressive effect. $+$ 1 represents that they can combine. This drug combination may produce a synergistic or additive effect. 0 indicates that they are unrelated. In the study, the observed drug combinations is denoted as *C*, where $c_{ij}=0$, if drug combination $(v^{[d]}_i,v_j^{[d]})$ is unobserved or unrelated in medical database, $\pm 1$ otherwise. $+$ 1 denotes the synergistic or additive effect, − 1 denotes the antagonistic or suppressive effect.

### Objective function

As shown in Fig. 1, the heterogeneous network is categorized into three classes, i.e., the drug–drug network, drug—protein network, and protein–protein interaction. Given the similarity matrix of drug network $S^{[d]}$, nonnegative matrix factorization (NMF) [44] is employed to extract the feature of drugs as

2 $$\begin{aligned} S^{[d]} \approx W^{[d]}H^{[d]}, \quad s.t. \quad W^{[d]}\ge 0, H^{[d]}\ge 0, \end{aligned}$$

where $W^{[d]}$ and $H^{[d]}$ are the basis and feature matrix, respectively. Equation (2) is solved by minimizing the approximation, i.e.,

3 $$\begin{aligned} \min \Vert S^{[d]}-W^{[d]}H^{[d]}\Vert ^{2}, \quad s.t. \quad W^{[d]}\ge 0, H^{[d]}\ge 0. \end{aligned}$$

Here, symmetric NMF (SNMF) [45] is performed to extract the features of drugs by exploiting the topology of $G^{[d]}$ since the similarity matrix is systematic. In this case, Eq. (3) is transformed the optimization problem as

4 $$\begin{aligned} \min \Vert S^{[d]}-W^{[d]}(W^{[d]})^{'}\Vert ^{2}, \quad s.t. \quad W^{[d]}\ge 0, \end{aligned}$$

where $W^{'}$ denotes the transpose of *W*. By minimizing Eq. (4), SeHNE generates a low dimensional feature vector for each drug, i.e., graph embedding.

Graph embedding for drugs in Eq. (4) is insufficient to fully characterize the features of drugs since the associations between drug and proteins are neglected. To address this issue, it is wise to extract features from the drug–target network. NMF is employed to extract the features of drugs and proteins, i.e.,

5 $$\begin{aligned} \min \Vert S^{[t]}-W^{[t]}H^{[t]}\Vert ^{2}, \quad s.t. \quad W^{[t]}\ge 0, H^{[t]}\ge 0, \end{aligned}$$

where $W^{[t]}$ and $H^{[t]}$ are the graph embedding for drugs and proteins under the drug-target network, respectively. However, features of proteins $H^{[t]}$ solely reflects the structure of drug—target networks without exploring the information of protein–protein interactions. The most intuitive strategy is to obtain features of proteins by factorizing $S^{[p]}$, i.e.,

6 $$\begin{aligned} \min \Vert S^{[[p]]}-H^{[p]}H^{[p]}\Vert ^{2}, \quad s.t. \quad H^{[p]}\ge 0. \end{aligned}$$

Then, we can combine Eqs. (5) and (6) to obtain graph embedding for proteins as

7 $$\begin{aligned} \Vert S^{[t]}-W^{[t]}H^{[t]}\Vert ^{2}+\Vert S^{[[p]]}-H^{[t]}H^{[t]}\Vert ^{2}, \quad s.t. \quad H^{[t]}\ge 0. \end{aligned}$$

However, the size of drug–target network is much less than that of protein–protein interaction networks, where Eq. (7) is dominated by the protein–protein interaction network. To address the problem, we adopt the regularization strategy to integrate protein–protein interaction network, where graph embedding for proteins $H^{[t]}$ must preserve the local topological structure in $G^{[p]}$. Luckily, the Laplacian regularization meets our expectation [46, 47], which is formulated as

8 $$\begin{aligned} \min tr((H^{[t]})^{'}L^{[p]}H^{[t]}) \end{aligned}$$

where $L^{[p]}$ is the Laplacian matrix for $G^{[p]}$. In this case, the features of protein–protein interaction network is transformed to graph embedding for drugs.

Notice that there are types of graph embedding for drugs either from drug–drug network or drug–target network. We aggregate them as the embedding of drugs. On the classification of drug combination, the loss function for binary classification is employed by mapping drug pairs with − 1 or $+$ 1, which is formulated as

9 $$\begin{aligned} {\mathcal {L}}^{[c]}(\phi ) = \sum _{c_{ij}=\pm 1}\ell ^{[hl]}(\phi (E_i,E_j),c_{ij}), \end{aligned}$$

where $\ell ^{[hl]}(\phi (E_i,E_j),c_{ij}) = max(0,1-c_{ij}\phi (E_i,E_j))$ is the hinge loss, $\phi (E_i,E_j)=\langle \theta ,K\left( E_i,E_j\right) \rangle$ is the inner product of $\theta$ and *K*, $E_{i}$ is the feature vector of drug $v_{i}^{[d]}$.

Finally, let ${\mathcal {L}}^{[d]}$ and ${\mathcal {L}}^{[t]}$ denote the loss function of two drug embeddings

10 $$\begin{aligned} \begin{aligned} {\mathcal {L}}^{[d]}&= \Vert S^{[d]}-W^{[d]}(W^{[d]})^{'}\Vert ^{2}\\ {\mathcal {L}}^{[t]}&= \Vert S^{[t]}-W^{[t]}H^{[t]}\Vert ^{2}+tr((H^{[t]})^{'}L^{[p]}H^{[t]})\\ \end{aligned} \end{aligned}$$

By combining Eqs. (4), (5), (8) and (9) to construct the joint learning framework for drug combination, we formulate the final objective function of the proposed algorithm as

11 $$\begin{aligned} \begin{aligned} {\mathcal {L}}(W^{[d]},W^{[t]},H^{[t]})&= {\mathcal {L}}^{[d]}+\lambda _1{\mathcal {L}}^{[t]}+\lambda _2{\mathcal {L}}^{[c]}\\&=\Vert S^{[d]}-W^{[d]}(W^{[d]})^{'}\Vert ^{2}\\&\quad +\lambda _{1}(\Vert S^{[t]}-W^{[t]}H^{[t]}\Vert ^{2}\\&\quad +tr((H^{[t]})^{'}L^{[p]}H^{[t]}))\\&\quad +\lambda _2\sum _{c_{ij}=\pm 1}\ell ^{[hl]}(\phi (E_i,E_j),c_{ij})\\&\quad \text {s.t.} W^{[d]}\ge 0,W^{[t]}\ge 0,H^{[t]}\ge 0, \end{aligned} \end{aligned}$$

where $\lambda _1$, $\lambda _2$ are parameters.

In the next subsection, we derive the updating rule to minimize the objective function in Eq. (11).

### Optimization rules

Equation (11) is non-convex, which can not be directly optimized. The iteration-based strategy is adopted, which updates one variable by fixing the others until the algorithm is convergent.

On the optimization of $W^{[d]}$, we aim to obtain the optimal matrix $W^{[d]}$ by fixing $W^{[t]}$ and $H^{[t]}$. By removing irrelevant terms to $W^{[d]}$ and employing the alternating minimization algorithms ANLS [48] to solve symmetric problem, the problem expressed in Eq. (11) is transformed into an optimization problem as

12 $$\begin{aligned}&\min \Vert S^{[d]}-W^{[d]}H^{[d]'}\Vert ^2+\lambda \Vert W^{[d]}-H^{[d]}\Vert ^2\\&\quad +\lambda _2\sum _{c_{ij}=\pm 1}\ell ^{[hl]}\left( \phi (E_i,E_j),c_{ij}\right) , \end{aligned}$$

The partial deriative on $W^{[d]}_i$ is derived as

13 $$\begin{aligned} \begin{aligned} \nabla _{W^{[d]}_i}&=\sum _{i,j}(W^{[d]}_iH^{[d]'}_j-s_{ij}^{[d]})H^{[d]}_j\\&\quad +\lambda (W^{[d]}_i-H^{[d]}_i)\\&\quad +\lambda _2\sum _{c_{ij}=\pm 1}\nabla _{W^{[d]}_i}\ell (\phi (E_i,E_j),c_{ij}), \end{aligned} \end{aligned}$$

where $\nabla _{W^{[d]}_i}\ell (\phi (E_i,E_j),c_{ij})$ is the gradient of hinge loss with respect to $W^{[d]}_i$.

On the optimization of $W^{[t]},H^{[t]}$, the problem for $W^{[t]}$ and $H^{[t]}$ are deduced as

14 $$\begin{aligned} &\min \lambda _1\Vert B^{[t]}-W^{[t]}H^{[t]'}\Vert ^2\\&\quad +tr(H^{[t]'}L^{[p]}H^{[t]})\\&\quad +\lambda _2\sum _{c_{ij}=\pm 1}\ell ^{[hl]}\left( \phi (E_i,E_j),c_{ij}\right) , \end{aligned}$$

The partial derivatives for $W^{[t]}$ and $H^{[t]}$ are calculated as

15 $$\begin{aligned} \begin{aligned} \nabla _{W^{[t]}_i}&=\lambda _1\sum _{i,j}(W^{[t]}_iH^{[t]'}_j-b_{ij}^{[t]})H^{[t]}_j\\&\quad +\lambda _2\sum _{c_{ij}=\pm 1}\nabla _{W^{[t]}_i}\ell (\phi (E_i,E_j),c_{ij}), \end{aligned} \end{aligned}$$

and

16 $$\begin{aligned} \nabla _{H^{[t]}}=\lambda _1(H^{[t]}W^{[t]'}W^{[t]}-B^{[t]'}W^{[t]}+L^{[p]}H^{[t]}) \end{aligned}$$

On the optimization of function $\phi$, we set it as the same strategy for the soft-margin linear SVM. The procedure of SeHNE is illustrated in Algorithm 1. 
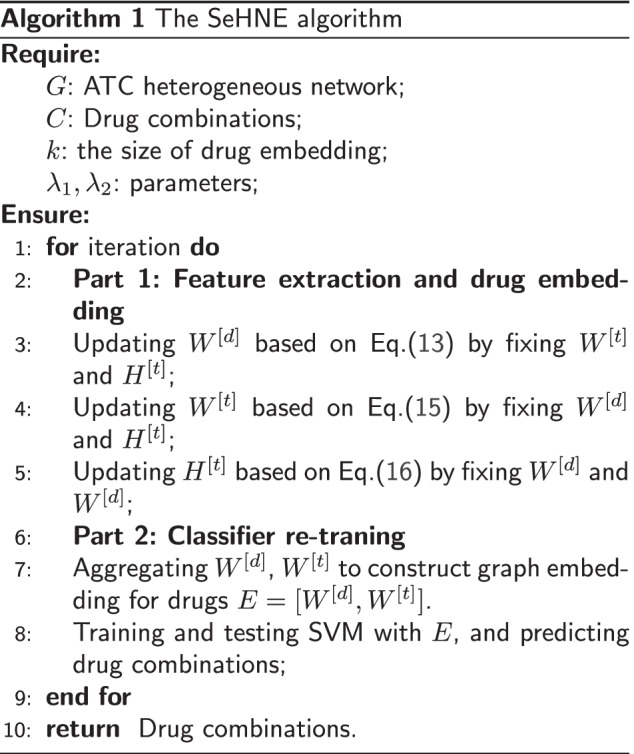


### Algorithm analysis

On the space complexity of the SeHNE algorithm, given a heterogeneous network, the space for ATC similarity network $O(n_d^2)$, the space for Drug-target network $O(n_dn_t)$, and the space for PPI network $O(n_t^2)$. The space for drug embedding and protein embedding are $O((n_d+n_t)k)$. Therefore, the overall space complexity is $O(n_d^2+n_t^2+n_dn_t+(n_d+n_t)k)=O((n_d+n_t)^2+(n_d+n_t)k)=O((n_d+n_t)(n_d+n_t+k))$. In our experiment, $k<n_d<n_t$. Therefore, $O((n_t+n_t)(n_t+n_t+n_t))=O(n_t^2)$, demonstrating that the proposed method is efficient in space complexity.

Then, the time complexity is analyzed. For each single-type or bipartite networks, SeHNE consists of three major components: symmetric NMF for similarity network, graph regularized NMF for Drug-target network and PPIs network, classifier learning for drug embeddings. The time complexity of updating $W^{[d]}$ is $O(r(n_d^2k+n^2k))$, where *n* is the number of known drug combinations, *r* is the number of iteration. The time complexity of updating $W^{[t]}$, $H^{[t]}$ are $O(r(n_t^2k+n^2k))$. The time complexity of updating SVM classifier is based on the number of support vectors, and much more fast than embedding extraction. So the overall time complexity is $O(r(n_d^2+n_t^2+n^2)k)$. In our experiment, $n_d<n_t<n$. Therefore, $O(r(n_d^2+n_t^2+n^2)k)=O(r(n^2+n^2+n^2)k)=O(rn^2k)$.

### Data

The training data for drug combination is downloaded from DrugCombDB [49] with the leukemia-related Cell lines. The drug-target interactions are derived from DrugBank, KEGG, and Therapeutic Target Database (TTD) datasets, covering 874 drugs and 1240 targets. Among them, we then removed a set of drugs, which don’t have enough information in the drug–drug network or drug–target network. Last, we obtained a group of 370 drugs and 18,126 drug–drug combinations. In general, there are 18,126 drug-drug combinations, consisting of 5903 synergistic and 12,223 antagonistic drug combinations. We summarize the fundamental properties of the drug–drug combinations network (Table 3). The PPI network of humans covers 15,911 proteins with 217,109 interactions [50].

**Table 3:** The analysis of heterogeneous network data

Scale	Entries	Value
Global	Number of DDIs	18126.00
	Number of drugs	370.00
	Number of synergistic DDIs	12223.00
	Number of antagonistic DDIs	5903.00
Drug degree	Average degree of drug	97.04
	Median degree of drug	119.50
	Maximal degree of drug	343.00
Synergistic effect drug degree	Average degree of drug	31.63
	Median degree of drug	24.00
	Maximal degree of drug	190.00
Antagonistic effect drug degree	Average degree of drug	65.72
	Median degree of drug	61.50
	Maximal degree of drug	208.00

### Criteria

A couple of performance measures were used in our experiment, including Accuracy, Precision, Recall, F-measure, Matthews correlation coefficient (MCC), and the area under the receiver operating characteristic curve (AUC). They are formally defined as below:

$$\begin{aligned} Accuracy&= \frac{TP + TN}{TP + FP + TN + FN}\\ Precision&= \frac{TP}{TP + FP}\\ Recall&= \frac{TP}{P} = \frac{TP}{TP + FN}\\ F-measure&= \frac{2 * Precision * Recall}{Precision + Recall}\\ MCC&= \frac{TP*TN-FP*FN}{\sqrt{P*N*(TP+FP)*(TN+FN)}} \end{aligned}$$

where *P*, *N*, *TP*, *FP*, *TN*, and *FN* are the numbers of real positives, real negatives, true positives, false positives, true negatives, and false negatives, respectively. The AUC is one of the most popular evaluation metrics [51]. AUC is the area under the receiver operating characteristic (ROC) curve, which plots the true positive rate (TPR) versus the false positive rate (FPR).

Based on the therapeutic organ or system of the drug, drugs are classified in the Anatomical Therapeutic Chemical (ATC) coding system. The ATC similarity between $v_{i}^{[d]}$ and $v_{j}^{[d]}$ is defined as:

17 $$\begin{aligned} S_{ij}^{ATC} =\frac{ATC(v_{i}^{[d]})\cap ATC(v_{i}^{[d]})}{ATC(v_{i}^{[d]})\cup ATC(v_{i}^{[d]})}. \end{aligned}$$

By observing the similarity matrix obtained from the above formula and the definition of ATC similarity, we can get that the ATC similarity matrix is sparse, which is advantageous for application in large-scale networks. On the other hand, the use of ATC as drug-similarity is limited, as the first level is anatomical so that a drug could be found across different body systems.

We also measured the Chemical Structure Similarity(CSS) of drugs to take different information of drugs into account. We get the smiles of each drug from the Drugbank database and then calculate the Molecular ACCess System (MACCS) fingerprints of drug molecules [52] according to its smiles by RDKit (https://github.com/rdkit/rdkit). MACCS is a binary fingerprint (zeros and ones) that answer 166 fragment-related questions. If the explicitly defined fragment exists in the structure, the bit in that position is set to 1, and if not, it is set to 0. Therefore, the drug fingerprint is a binary sequence. Then, the Jaccard similarity method is employed to calculate the chemical structure similarity of drug–drug pairs based on molecular fingerprints. Let *A* and *B* represent the counts of bits in the two-drug molecules, respectively, the chemical structure similarity between set (drug) *A* and *B* is defined as follows:

18 $$\begin{aligned} S_{ij}^{CSS} = J(A,B) = \left| \frac{A\cap B}{A\cup B} \right| = \frac{\left| A\cap B \right| }{\left| A \right| + \left| B \right| - \left| A\cap B \right| } \end{aligned}$$

To utilize the information of two kinds of similarity matrices, two drug-drug similarities are integrated into a comprehensive similarity measure by the probability disjunction formula as

19 $$\begin{aligned} S_{ij}^{ATC \& CSS} = 1 - (1 - S_{ij}^{ATC})(1 - S_{ij}^{CSS}) \end{aligned}$$

$S_{ij}^{ATC}$ is the ATC similarity between $Drug_{i}$ and $Drug_{j}$ , $S_{ij}^{CSS}$ is the chemical structure similarity between $Drug_{i}$ and $Drug_{j}$.

## Data Availability

The data are publicly available in DrugCombDB (http://drugcombdb.denglab.org/), Drugbank (https://go.drugbank.com/), and TTD (http://db.idrblab.net/ttd/).

## Abbreviations

SVM: Support vector machine
TCGA: The Cancer Genome Atlas
FPKM: Fragments Per Kilobase of transcript per Million fragments mapped
COSMIC: The Catalogue Of Somatic Mutations In Cancer
NMF: Nonnegative matrix factorization
